# Paving the Way Towards a Successful and Fulfilling Career in Computational Biology

**DOI:** 10.1371/journal.pcbi.1003593

**Published:** 2014-05-01

**Authors:** Olugbenga Oluwagbemi, Marion Adebiyi, Segun Fatumo, Geoff Macintyre

**Affiliations:** 1 Department of Computer and Information Sciences, College of Science and Technology, Bioinformatics Unit, Covenant University, Ota, Ogun State, Nigeria; 2 H3Africa Bioinformatics Network (H3ABioNet) Node, National Biotechnology Development Agency (NABDA), Federal Ministry of Science and Technology (FMST), Abuja, Nigeria; 3 Human Genetics Department, Wellcome Trust Sanger Institute, Cambridge, United Kingdom; 4 International Health Research Group, Department of Public Health and Primary Care, University of Cambridge, Cambridge, United Kingdom; 5 Centre for Neural Engineering, The University of Melbourne, Carlton, Victoria, Australia; 6 Department of Computing and Information Systems, The University of Melbourne, Parkville, Victoria, Australia; Princeton University, United States of America

## Introduction

Most of us will spend a significant amount of time and effort throughout our lives in improving our career. The decisions we make shape how our career progresses, and the right decisions can ensure it is successful and fulfilling. Early decisions can have a strong influence, especially in today's competitive job market, where a university degree will not guarantee the best job. It is vital these early decisions are well informed and based on access to as much information as possible. As part of an effort to ensure that computational biologists and students are guided into the right career paths, the Regional Student Group (RSG) program, an arm of the International Society for Computational Biology (ISCB), has provided a range of activities to assist computational biologists and bioinformatics researchers in their career development. These include organizing practical workshops and seminars presented by leading experts on how to broaden the scope of career options and guarantee success. This article provides insight on some of these activities and highlights the benefits gained through the shared experiences of RSGs in running career-related activities.

## The Importance of Career Development

It is important to realize the broad impact of a job on an individual. A good career path can improve quality of life, create a sense of purpose, provide adequate income, and determine the social and economic contributions an individual can make to their community and society. In order to have a successful and fulfilling career, it is beneficial to take part in developmental activities which not only open doors to the right career but also develop awareness and insight into how career choices taken now can affect the future. Appropriate career development activities can therefore be as simple as making sure a Curriculum Vitae (CV) is formatted correctly, or as involved as attending a seminar on how a particular field is expected to develop and how to tailor career choices accordingly. In many cases, a typical science-based university degree needs to be supplemented with activities that help develop a student's career options. In computational biology, there are a number of activities designed with this in mind. For instance, the Wellcome Trust advanced courses provide opportunities for young researchers from Africa to attend international workshops, conferences, and other research training in the United Kingdom. The European Molecular Biology laboratory also provides good career development opportunities in the form of fellowships, research grants, and scholarships to train African and European students. However, for students who are not eligible for these programs, a number of RSGs have undertaken initiatives to provide career development opportunities, some of which are outlined below.

## RSG Career Development Activities

RSGs have organized a range of activities aimed at career development. While diverse, they can be broadly grouped into two categories: events providing information on career opportunities and events providing information on how to market yourself and improve your chances of getting the right job. In both categories, the RSG was attempting to address an area of deficiency and improve overall career prospects.

The workshops run by RSGs range from introductory career workshops enticing students to consider a career in the field of computational biology to advanced workshops for those already developed in the field but striving for more. For instance, for those new to computational biology, RSG Argentina participates in annual career talks held at Universidad Nacional de Entre Rios, where they provide personal insights to prospective students who aspire to take up bioinformatics and computational biology. In this case, direct contact with someone in the field reduces the barrier to entry, as a cross-disciplinary field such as computational biology can be daunting, and a fresh-faced student can help remind others of the benefits and exciting prospects of entering the field.

RSG France has tackled the next career stage by running the JOBIM workshop “Starting as a Bioinformatician.” This event provides students with handy hints on what to expect when entering the field, and gives them a series of prerequisites which would set them in good standing from the outset. Similarly, in 2011, RSG Central Africa organized a Central African Regional Bioinformatics workshop on principles and potentials in bioinformatics. The practical tips provided at these events ensure that a student can be confident in embarking on their chosen career path.

For help on the way, RSGs have also provided members with information about scholarships and fellowships. For example, RSG Eastern Africa engaged an expert to give a seminar about Commonwealth Scholarship opportunities and tips on how to boost students' chances of receiving a scholarship. At this event, a number of current RSG members provided accounts of their success in acquiring one of these scholarships.

A good balance between specific career advice and broad career planning has been achieved across the RSGs, as in RSG France's JeBiF satellite meeting, which communicated career opportunities in bioinformatics within France, and RSG Netherland's retreat in 2012, where Professor Phil Bourne gave a talk about his career path and the keys to his successes. All of these workshops provide a great resource for students to broaden their knowledge about future career prospects.

Other activities run by RSGs have focused on the marketing and soft-skills aspect of career development. Many RSGs have run student symposiums which provide a mechanism for students to improve their networking and presentation skills. RSG Europe took this one step further: at the first European Student Council Symposium, they employed an actor-turned-scientist to deliver a hands-on workshop on the art of presenting science. RSG France also looked to outside experts when they held a career session on developing and designing a personal webpage. A number of the best webpages were critiqued and used as examples to teach members how to improve their own web pages. Without a doubt, the most common career-related question asked by RSG members relates to writing motivation letters, CVs, and personal statements for scholarships. A number of RSG activities have focused around helping students improve these, particularly by understanding what selection committees are looking for in an applicant's CV. RSG-France's annual meeting centres on this topic and has proven to be successful across the board.

These RSG career development activities are designed to supplement canonical training and provide students with a well-rounded education and good standing for their future careers.

## How to Run a Career Development Activity

The most important factor in running a successful career development activity is identifying a relevant topic [1]. This usually involves finding a balance between what student members want and what employers would like to see. Therefore, it is important to quiz both students and employers in the chosen area in order to identify a beneficial activity. It is also important to ensure that a similar opportunity is not offered elsewhere and that a real gap in attendees' knowledge is to be addressed. For example, if their degree already includes a course on assessing the current state of the job market, it is unlikely a student-run event on the same topic will add anything significant. Effort is best directed at addressing an area which is not dealt with elsewhere.

Once the topic has been chosen, it is crucial to put together a good plan. This includes finding a good speaker/facilitator (if needed), acquiring the necessary resources, securing a venue, and making sure the event is well advertised. Once all these bases are covered, you should be well prepared to hold a successful career development event. However, not all things will go according to plan, so spending time on identifying where things might go wrong and putting contingency plans in place is vital. Without these, problems may escalate. For example, it is important to correctly estimate the costs of an event, particularly when applying for funding well in advance; however, costs can increase from when the budget is first written to the time the event is staged.

In one unfortunate example, an RSG was unable to run their planned career development event—in this case, we can identify where things could have been organized differently, to help ensure the same situation does not arise with future events. The problem arose when funds were required to run an event planned by RSG West Africa. At the time of submitting the proposal, they carefully estimated costs to maximize what they could achieve on a tight budget. Unfortunately, due to political unrest, the event had to be rescheduled and relocated. This meant that the costs increased and the initial funding grant was not enough. The event was designed such that each aspect was critically dependent on the funding in order to go ahead. Therefore, nothing could be cut, and the event could not go ahead. In this case, it would have been helpful to have some flexibility in the budget to deal with potential increases in costs. A good budget should really include two cost estimates: one which identifies the bare minimum to run the event and another which provides for a fully featured event. If the original funding proposal is based on the latter, the alternate costing would provide flexibility to run the event if costs increase, in which case, some of the noncritical features could be dropped.

## Conclusion

We have provided a snapshot of career development events run by RSGs and identified their benefits, with the desire to encourage others to run similar activities. These events have provided a forum where RSG members are guided into profitable future careers. They can provide a platform to inform students about diverse opportunities in computational biology and bioinformatics and how to harness these opportunities. However, one of the greatest career development opportunities gained through these activities is in the planning and running of the event itself. A wealth of soft skills are obtained, such as good and effective communication, good human relations, efficient planning skills, and effective networking. The importance of running and participating in these activities cannot be overemphasized.

**About the Authors**

The authors have been involved in organising various events for Regional Student Groups and the ISCB Student Council. **Olugbenga Oluwagbemi** is the current vice president for RSG Western Africa (2013–present). **Marion Adebiyi** was the president of RSG Western Africa (2008–2012). **Segun Fatumo** was founder and president of RSG Africa (2007–2009), and is currently vice chair of the RSG WorldWide program (2011–present). **Geoff Macintyre** served as chair of the ISCB Student Council (January 2011–January 2013) and was co-founder and president of RSG Australia (2009–2010).
